# The Relation between the Physician and Pharmaceutist

**Published:** 1872-08

**Authors:** Z. T. Daniel

**Affiliations:** Eufaula, Alabama


### THE RELATION BETWEEN THE PHYSICIAN AND PHARMACEUTIST.

[*REPLY TO TH. SCHUMANN, PHARMACEUTIST, ATLANTA, GA.*]

BY Z. T. DANIEL, M.D., EUFAULA, ALABAMA.

In the JOURNAL for May, 1872, my attention was directed to an article bearing the above caption. Through the whole article, it is shown that the intentions of Mr. Schumann are to do right, by elevating the vocation of the prescriptionist and in dignifying the medical profession.

I shall not attempt to reflect upon the judgment of Mr. Schumann; but representing, as he does, the cause of the pharmaceutist or druggist, I propose to elucidate broad and palpable quackery practiced by no regular physician, but unremittingly prosecuted by every druggist in whose store I ever had occasion to put my foot.

Mr. Schumann remarks, that "the best guard, therefore, for both physician and the pharmacist, against quackery of any kind, would be the enforcement of a proper law regulating the practice of medicine and pharmacy respectively."

Now, as to regulating the practice of *medicine* by law, I will simply state that such a hope is a treacherous delusion, and I think the gentleman, upon reflection, will see that it would be just as successful as an attempt to regulate the practices in the sale of produce and fabrics, which are not regulated by law, but by consent of salesmen, supply and demand in the markets.

I consider that one of the important steps toward medical reformation is the annihilation of all patented nostrums, together with the system of their manufacture and sale, which are put upon the ignorant people of our country by the scum, scoria, and outlaws of the regular medical profession. Who are instrumental? What is the patent lever by which these specifics (so-called) are raised from professional prostitution and mercenary baseness, and furnished with a show of respectability? Where is the spacious avenue through which these plagues to humanity invitingly course their death-bound march? The answers cluster around every drug-store in the land, while fair Science, imprisoned and chained within, exclaims, "Here! here!" I say that in this trade with patented or proprietary medicines, nostra and all kindred calamities, the druggist is unquestionably the most formidable enemy to his own interests, and to the advancement of the most humane and peerless science within the cycle of time.

Let the druggist cease to trade in these things; let representatives and *wise* senators be *taught* and *lessoned*, and thereby secure legislation against their baneful influence upon the cause of science—their deleterious effect as misdirected to suffering humanity; and then physician and druggist will begin to prosper, and science will advance apace. Physicians have no desire to associate with you until you lend a helping hand to cut down this national monster, which has its existence mainly in your hands, and which is so deadly to the best interests of their profession and their purse.

As to whom a written prescription belongs, it seems to me that it is the property of physician, patient and druggist, jointly, and neither can claim exclusive ownership—the physician needing it to demonstrate what he prescribed, the druggist what he compounded, and the patient to show what was administered. In relation to repeating it without instructions from the physician, no intelligent pharmaceutist would do it; but a blunderer and botcher will take the responsibility upon himself to re-prescribe, under an opinion that the physiological necessities of the patient require a repetition of the identical prescription. Many such persons, I am sorry to say, occupy the position of pharmaceutist. However, we will not make any important issue about that sort of medication, if the pharmaceutist will agree to be sued for practicing medicine without license; and as the physician would not be known in that consultation, of course, no responsibility could be attached to him.

In regard to physicians dispensing their own medicines, the remarks of Mr. Schumann are well-timed, but wholly inapplicable to those representatives of the profession in the villages and rural districts.

If well-educated people give themselves entirely up to the skillful physician, and uneducated ones do not, then it becomes Mr. Schumann and his colaborers to educate these unfortunate ones, and not feed and pamper their lamentable condition by taking charge partly themselves.

I would most courteously and respectfully ask Mr. Schumann to institute, if possible, some means, in the next meeting of the American Pharmaceutical Association, relative to the prohibition of the universal sale of already-compounded humbugs, purporting to be all-healing prescriptions, denounced by the medical profession, and by every intelligent scientist who ought to know, by which they may be uprooted and removed from the paths of our profession. Then will the science of medicine and pharmacy roll on with unclogged wheels until they reach that final goal of all our expectations—perfection.

